# Evaluation of a Diagnostic Therapeutic Educational Pathway for Asthma Management in Children and Adolescents

**DOI:** 10.3389/fped.2020.00039

**Published:** 2020-03-11

**Authors:** Sebastiano Guarnaccia, Cristina Quecchia, Andrea Festa, Michele Magoni, Elena Zanardini, Valentina Brivio, Carmelo Scarcella, Valeria Gretter, Cinzia Gasparotti, Malica Frassine, Martina Ferrari, Rosa Maria Limina, Raffaele Spiazzi, Raffaele Badolato, Francesco Donato

**Affiliations:** ^1^Department of Clinical and Experimental Sciences, University of Brescia, Brescia, Italy; ^2^“Io e l'Asma” Center, Ospedale dei Bambini, ASST Spedali Civili, Brescia, Italy; ^3^Department of Medical and Surgical Specialties, Radiological Sciences and Public Health, University of Brescia, Brescia, Italy; ^4^Brescia Health Protection Agency, Brescia, Italy; ^5^Post-graduate School of Public Health, University of Brescia, Brescia, Italy; ^6^Insubria Health Protection Agency, Varese, Italy; ^7^I.R.C.C.S. Giannina Gaslini, Genova, Italy

**Keywords:** asthma, asthma management, children and adolescents, GINA guideline adherence, clinical educational pathway, real-life research

## Abstract

**Background:** Limited evidence exists for the effectiveness of educational programs that improve pediatric asthma control in real-world settings. We aimed to assess the impact of a diagnostic, therapeutic, and educational pathway (DTEP) for asthma management in children and adolescents attending an asthma referral center.

**Methods:** This is a retrospective population-based cohort study, including two groups of patients with asthma, aged 6–17 years and residing in the Local Health Authority (LHA) of Brescia, Italy: (a) the children who followed a DTEP (intervention group) and (b) all the children residing in the LHA who did not follow DTEP (control group). The incidence rates (IRs) of hospitalization, emergency room visit, use of outpatient services, and drug prescription for dyspnea, wheezing, or respiratory symptoms were computed for time before and after attending DTEP in the intervention group and for “early” and “late” time since asthma diagnosis in the control group.

**Results:** There were 9,191 patients included in the study, 804 of whom followed DTEP. In the before-DTEP/early time, the intervention and control groups showed similar IRs for all the outcomes apart from emergency room visits (IRs of 138.6 and 60.3 per 1,000 person-years, respectively). The IRs decreased from before to after DTEP and from early to late time in both groups. The IR decrease for emergency room visits was significantly higher in the intervention than in the control group (−51.3 and −28.2%, respectively; IRR = 0.61, *P* = 0.001).

**Conclusion:** The DTEP can increase patients' capability in managing asthma and preventing asthma attacks.

## Introduction

Asthma is the most common chronic disease among children. Childhood asthma is usually defined as a heterogeneous disease, usually characterized by chronic airway inflammation. It is defined as an umbrella syndrome, with symptoms such as wheeze, shortness of breath, chest tightness, and cough that vary over time and in intensity, together with variable expiratory airflow limitation (1–3). The long-term goals of asthma management are to achieve symptom control, to minimize future risk of asthma attacks and airflow limitation, to preserve lung function, and to reduce the risk of adverse effects of treatment (1). Asthma control regards two different domains: symptom control and risk factors for future asthma attacks (1). Symptom control regards daytime (daytime symptoms more than twice a week) and nighttime (any night waking due to asthma) symptoms, reliever use (reliever needed more than twice a week) for symptoms treatment, and deviation from normal activity (any activity limitation due to asthma). Future risk of poor asthma outcomes domain includes preventing severe asthma attack, loss of lung function, and adverse effects caused by medication use (1–3).

Adherence to guideline-based care is challenging for various reasons (4, 5). Clinicians do not often follow current guidelines and also express lack of confidence in the ability of patients to adhere to their recommendations, coupled with clinical inertia, general practice barriers, and time constraints (6–8). Overall, a substantial gap exists between the actual care provided for pediatric asthma and the recommendations in national guidelines (9).

Some randomized controlled trials (RCTs) showed that interventions for educating children in various settings, particularly children attending emergency department for asthma, can be effective for asthma control (10–12). A randomized trial on the effectiveness of an educational program among primary care providers showed that patients of physicians who attended the program had a greater decrease in days limited by asthma symptoms and decreased emergency department visits (13). However, although patient-centered education programs are considered the key factor for the success of asthma self-management (5, 10), few studies exist on the impact of patient-oriented asthma education programs based on the comparison between patients participating and those non-participating in these programs in the real world (14).

In the present study, we aimed to assess the impact of a diagnostic, therapeutic, and educational pathway (DTEP) for asthma management in children and adolescents, provided by a referral center, through the comparison between patients attending and non-attending the center, using objective, routinely collected, outcome asthma measures.

## Methods

“Io e l'Asma” is an outpatient pediatric asthma center established in 2003, which provides patients with a DTEP for asthma management to achieve a better control of the disease and prevent asthma attacks and adverse outcomes, through a strong cooperation between clinicians (specialists and primary care physicians) and therapeutic educators (15). Asthma diagnosis was based on clinical history and spirometry with reversibility test, according to the Global Initiative for Asthma (GINA) diagnostic criteria for adolescents and children (1).

The DTEP is a multidisciplinary approach that includes three specialist examinations, every 8–12 weeks, and two follow-up visits 6 and 12 months after the last specialist examination, as described in detail elsewhere (15, 16). Briefly, the specialist evaluations include first a clinical and instrumental examination, with an overall assessment of patients and possible changes in asthma treatment. Second, an educational session is provided for teaching patients and their parents how to prevent and manage asthma attacks, identify symptoms quickly, use drugs and devices correctly, maintain a healthy lifestyle, and keep a diary for monitoring clinical features, by a health care assistant.

After each specialist evaluation, patients are sent to their primary care pediatricians for monitoring clinical conditions and checking asthma control and daily therapy.

This is a retrospective population-based cohort study, including two groups of patients with clinically diagnosed asthma, aged 6–17 years and residing in the Local Health Authority (LHA) of Brescia, Lombardy region, Italy: (a) the subjects who followed the DTEP (intervention or DTEP group) and (b) all the children residing in the LHA who never joined the DTEP program (control group). All subjects were followed between September 1, 2007, and December 31, 2014.

Control children and teenagers were identified in the LHA database using the following criteria:

a) *International Classification of Diseases, Ninth Revision*, codes of chronic pulmonary disease
b) Regional code of exemption from payment for asthma

Asthma diagnosis was made in these children during hospitalizations, accesses to outpatient services, emergency room visits or specialist' s evaluations, with spirometry and reversibility tests, prick and immunologic tests, and officially certified with the attribution of the asthma-exemption code.

The study was approved by the Ethics Committee of the “Spedali Civili di Brescia,” Brescia (Italy), on June 15, 2015 (registration no. 2046).

To analyze the impact of the DTEP, we compared the occurrence of the following asthma-related outcomes in both the intervention and control groups:

a) *hospitalization* with primary discharge diagnosis of dyspnea, wheezing, or respiratory symptoms;
b) *use of outpatient services* [spirometry, skin prick test, ImmunoCAP or microarray, total and specific immunoglobulin E (IgE)];
c) *emergency room visits* with primary discharge diagnosis of dyspnea, wheezing, or respiratory symptoms;
d) *drug prescriptions*, including the prescription of medicines for asthma by the children's primary care pediatrician; we used the World Health Organization international Anatomical Therapeutic Chemical Classification System to identify the categories of drugs used for asthma treatment (17).

The beginning of asthma was established at the date of the first occurrence of any asthma-related event in the study time (hospitalization, access to outpatient services, emergency room visit, drug consumption, or attribution of the asthma-exemption code). Therefore, only patients with the first asthma-related event during the observation time were included in the analysis (incident cases). Children with clinical diagnosis of asthma before the beginning of the study period (September 1, 2007) were excluded from the analysis.

The incidence rates (IRs) of asthma-related events were computed as the number of events per 1,000 person-years. The person-years were computed as the sum of the observation times from the asthma onset until the end of the observation time. The 95% confidence intervals (95% CIs) of the IRs were computed according to Poisson distribution.

**Figure 1:**
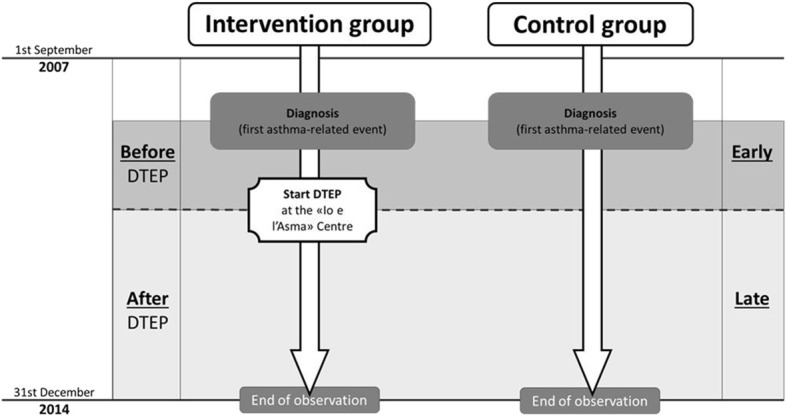
The two study times considered for computing incidence rates of health outcomes in patients attending (intervention group) or not attending (control group) a diagnostic, therapeutic, and educational pathway (DTEP): time before and after DTEP (left side) for the intervention group, and “early” and a “late” time since asthma diagnosis for the control group (right side).

Among patients attending the center (intervention or DTEP group), the IRs of each outcome were computed before (before DTEP) and after (after DTEP) attending the DTEP (Figure 1, left side). Similarly, an approximate time demarcation was made in the control group, using the average interval time between the dates of asthma diagnosis and of first attending the DTEP in the intervention group as the cutoff (1.5 years). Therefore, in the control group, the time between beginning of asthma and this cutoff was considered as “early” and the following time as “late” for each patient, and the corresponding IRs for each outcome in the early and late times were computed (Figure 1, right side).

The incident rate ratios (IRRs) were computed using a Poisson regression for repeated measures, which included as independent variables: age, gender, time of observation (before/after DTEP or early/late time since asthma diagnosis), group (intervention/control), and an interaction term between the last two variables. The IRRs for the interaction term between time of observation and group can be interpreted as indicative of the relative change of the IRs from before to after DTEP, or from early to late time since asthma diagnosis, in the intervention compared to the control group.

All the statistical tests were two-sided with *P* = 0.05 as the threshold for refusing the null hypothesis. The data analyses were performed using the Stata program for personal computer, version 14 (18).

## Results

A total of 9,191 patients were included in this study, 804 of whom followed DTEP. Among children in the intervention and control groups, similar proportions of subjects were resident in Brescia (17.4 and 14.6%, respectively) or in the rest of the province (82.6 and 85.4%, respectively). Age at asthma diagnosis was 9.29 and 11.13 years in the intervention and control groups, respectively.

Lower rates of asthma diagnostic tests were performed in the intervention than in the control group in the whole period: the IRs of spirometry with challenge test were 17.6 and 22.2 per 1,000, and those of reversibility test were 17.1 and 28.5 per 1,000, in the intervention and control groups, respectively. On the contrary, the IRs of allergy tests were higher in the intervention than in the control groups: the IRs for prick test were 174.8 and 59.2 per 1,000, and those of IgE assay were 77.9 and 73.9 per 1,000, in the intervention and control groups, respectively.

Considering the hospital discharge data, the follow-up time was 3,976.2 person-years for patients following the DTEP (3,199.1 and 777.1 person-years for 6–11- and 12–17-year-old children, respectively) and 42,355.6 person-years for those who did not follow the DTEP (25,429.8 and 16,925.8 person-years for 6–11- and 12–17-year-old children, respectively). The mean follow-up time for children attending the Io e l'Asma center was 1.50 years before DTEP and 3.53 years after DTEP. Similarly, for the control children, the mean follow-up was 1.50 and 3.68 years in the early and late time since asthma diagnosis, respectively.

The numbers and IRs of hospitalizations, emergency room visits, drug prescriptions, and use of outpatient services, according to study time, are set out in Table 1. In the first part of the study time, the two groups showed similar IRs for all the outcomes apart from emergency room visits, for which a more than double IR was observed in the former than the latter group (138.6 and 60.3 per 1,000 person-years, respectively). A decrease of all the IRs was observed from the first to the second part of the study time in both groups. However, a greater reduction of the IR of emergency room visit was found in children who followed DTEP program than in those who did not (control) (−51.3 and −28.2%, respectively), and therefore a lower risk in the former than the latter was observed (IRR = 0.61, *P* = 0.001). On the contrary, the IR of using outpatient services decreased less in patients attending DTEP compared to those who did not (−8.9 and −31.3%, respectively). No difference was found between the two groups in the IRs for hospitalization and drug prescription.

**Table 1:** Incident rates (IRs), percentage difference between after and before following a diagnostic, therapeutic, and educational pathway (DTEP) and between late and early time since asthma diagnosis, in the intervention and control groups, respectively, and incidence rate ratios (IRRs) for hospitalizations, emergency room visits, outpatient services, and drug prescriptions.

**Outcomes**	**Intervention group**					**Control group**					**IRR (95% CI) intervention to control group**	***P***
	**Before**		**After**		**% Difference after–before rates**	**Early**		**Late**		**% Difference late–early rates**		
	**No**.	**IR per 1,000** **(95% CI)**	**No**.	**IR per 1,000** **(95% CI)**		**No**.	**IR per** **1,000 (95% CI)**	**No**.	**IR per 1,000** **(95% CI)**			
Hospitalization	54	53.4 (38.0–68.8)	51	17.2 (11.2–23.2)	−67.8 (51.9 to 78.5)	824	52.3 (47.5–57.1)	443	16.7 (14.1–19.3)	−68.1 (64.2 to 71.7)	0.88 (0.56–1.39)	>0.1
Emergency room visit	116	138.6 (108.6–168.7)	181	67.5 (53.8–81.2)	−51.3 (38.0 to 61.6)	425	60.3 (53.3–67.3)	1,355	43.3 (40.3–46.3)	−28.2 (19.7 to 35.6)	0.61 (0.45–0.82)	0.001
Outpatient service	258	308.4 (263.6–353.1)	753	280.8 (256.8–304.8)	−8.9 (5.3 to 21.0)	1,734	246.8 (231.6–262.0)	5,314	169.7 (162.2–177.1)	−31.3 (27.4 to 34.9)	1.79 (1.50–2.14)	< 0.001
Drug prescription	7,873	7,846.1 (7,244.2–8,448.0)	12,911	4,488.1 (4,139.9–4,836.4)	−42.8 (41.2 to 44.4)	68,055	8,011.3 (7,830.7–8,191.9)	153,544	4,537.5 (4,404.8–4,670.3)	−43.4 (42.8 to 43.9)	0.97 (0.94–1.01)	0.51

*The incidence rate ratios for the intervention compared to the control group corresponded to the interaction term between the group and time since asthma diagnosis (before/after DTEP or early/late time) fitting a Poisson regression model for repeated measures including age, gender, and main effects for time and group*.

The numbers and IRs of prescription of each pharmacologic class used in asthma management according to study time are set out in Table 2. In the first part of the study time, the most commonly prescribed medications were salbutamol, inhaled corticosteroids, and antibiotics in both groups, with moderate differences between them, apart from antibiotics: the IR of antibiotics prescription was higher in the intervention than in the control group. A reduction of the prescription IRs from the first to the second part of the study time was observed for each drug in both groups. A statistically significantly lower risk of prescription of long-acting beta-agonists (LABA) plus glucocorticoid, leukotriene receptor antagonist, and antibiotics and a higher risk of prescription of salbutamol and inhaled corticosteroids were found in the intervention compared to the control group.

**Table 2:** Incident rates (IRs) and incidence rate ratios (IRRs) for use of main pharmacologic classes for asthma treatment.

	**Intervention (DTEP) group**				**Control (LHA) grou**				**IRR (95% CI) intervention to control group**	***P***
	**Before**		**After**		**Early**		**Late**			
**Drug class**	**No**.	**IR per 1,000**	**No**.	**IR per 1,000**	**No**.	**IR per 1,000**	**No**.	**IR per 1,000**		
LABA plus glucocorticoid	577	575	689	239.5	6,088	716.7	20,785	614.2	0.49 (0.43–0.54)	< 0.001
Salbutamol	1,538	1,532.8	2,951	1,025.8	11,776	1,386.2	27,593	815.4	1.14 (1.07–1.21)	< 0.001
Inhaled corticosteroids	1,595	1,589.5	2,571	1,181.7	14,466	1,702.9	22,980	679.1	1.70 (1.60–1.81)	< 0.001
Leukotriene receptor antagonist	487	485.3	677	235.3	7,687	904.9	19,886	587.7	0.75 (0.66–0.84)	< 0.001
Systemic steroids	418	416.6	617	214.5	3,628	427.1	7,358	217.4	1.01 (0.89–1.15)	0.866
Antibiotics	3,258	3,246.9	4,872	1,693.6	24,410	2,873.5	54,942	1,623.6	0.92 (0.88–0.97)	0.001

*The IRRs for the intervention compared to the control group corresponded to the interaction term between the group and time since asthma diagnosis (before/after DTEP or early/late time) fitting a Poisson regression model for repeated measures including age, gender and main effects for time and group*.

The most commonly used medications for asthma treatment in both groups are reported in Supplementary Table 1. The fluticasone/salmeterol combination was the most used LABA and glucocorticoid combination in both groups, with a higher reduction from before to after DTEP in the intervention than from early to late time in the control group. Montelukast is the only leukotriene receptor antagonist prescribed in Italy. Among systemic steroids, the most used ones were betamethasone and prednisone, and their IRs reduced in both groups similarly. Salbutamol was widely used (alone or, rarely, in combination with ipratropium bromide) for the treatment of asthma attacks, with a reduction of the IRs for this prescription from before to after DTEP and from early to late time, in the intervention and control groups, respectively.

## Discussion

This study showed that the IRs decreased in both intervention and control groups, from before to after DTEP time. However, the IR of emergency room visits declined more in the intervention than in the control group (−51 vs. −28%, respectively, IRR = 0.61), whereas the IR of use of outpatient service declined in the control group only. No difference was observed for hospitalization and drug prescription rates between the two groups.

The attendance in the emergency department is usually considered a valuable indicator of asthma control. Indeed, RCTs evaluated the risk of subsequent emergency department visits as health outcomes as a measure of the efficacy of asthma education interventions for improving asthma management (10, 11). Furthermore, a US RCT showed a significantly higher reduction of emergency department asthma visits in children whose physicians attended an educational seminar than those whose physicians did not (13). Noteworthy, the same trial showed no difference in asthma office visits and in hospitalizations for asthma between the intervention and control groups (13). In our study, the IR of emergency department room visits was initially higher in the intervention than in the control group. This finding suggests a spontaneous selection of children attending the center compared with those who did not, with a poorer control of asthma symptoms in the former, on the average, probably due to ineffective, or no treatment of asthma. Therefore, the higher reduction of the IR of emergency department visits in the intervention group suggests an overall positive impact of the program on asthma control. However, the DTEP included both specialist's visits and the children's and parents' educational intervention, which were closely linked together, as the effect of each of them cannot be disentangled.

The lack of decrease of outpatient service rate from the first to second time in the DTEP group, contrary to what was observed in the control group, was expected, as it is mainly due to the follow-up visits and monitoring tests (particularly spirometry and skin prick test or specific IgE) included in the DTEP itself (15–17, 19).

The rate of asthma drug prescription decreased in both groups, but with important differences in medication. First, the use of salbutamol and inhaled glucocorticoid decreased less in the intervention than in the control group, in line with present recommendations, as salbutamol is a reliever asthma drug that every patient should have with an action plan to prevent and to treat the asthma attack, and inhaled glucocorticoids are used to manage asthma in the first-line treatment (1). Second, the reduction of rate of prescription of LABA plus glucocorticoid and leukotriene receptor antagonist, as that of antibiotics, was higher in the intervention than in the control children, suggesting a better disease control with only inhaled corticosteroid and a lower rate of infections in the former than in the latter. Really, an increasing control of asthma symptoms, according to the assessment criteria of the GINA guidelines (1), was evident in children following the DTEP: the percentages of children with well-controlled asthma increased from 35.9% at the first visit to 82.5%, at the third visit, as reported previously (19). At present, a high proportion of subjects with uncontrolled asthma are still reported also in developed countries. Indeed, a European survey showed that 45% of respondents had uncontrolled asthma (20), and an international survey among adults and adolescents found that only 9% (range, 0–29%) had well-controlled asthma (21).

In this study, we could not assess the direct and indirect costs of asthma management; however, the decrease of IRs of emergency room visits and of asthmatic drug prescription suggests a decrease in both direct and indirect asthma-related costs. A computation of the cost of drug prescriptions for asthma was performed for 262 children aged 6 to 15 years attending the center, which showed a decrease of approximately 48% from before to after DTEP (16). This agrees with a long-term Finnish experience showing that improvement of asthma care determined a reduction in total costs for the disease, including costs of medication (22).

This study has notable strengths. First, it is a population-based study that compares the incidence of some objective asthma-related outcomes in the children who followed, and those who did not, a DTEP on asthma control, as a real-world research study. All the children and adolescents living in the area were registered in the health care database of the LHA, according to the Regional Health Service practices. Therefore, virtually all the children and adolescents who were resident in the area were included in the analysis, avoiding any selection bias: the children who followed the DTEP were included in the intervention group, and all the others, who never followed the DTEP, were in the control group. Second, because of the use of routinely collected administrative data included in the health care database of the LHA, we are confident that no substantial information bias occurred.

The study has some limitations, too. First, it has a retrospective cohort design, analyzing the impact of the DTEP in the past years. Second, the children attending the DTEP had more severe and less controlled asthma when they first attended the center, as shown by higher IRs for all the health outcomes in the intervention than in the control group, in the before DTEP and early time since asthma diagnosis, respectively. In fact, a high proportion of subjects with uncontrolled asthma were found at their first visit at the center (19). Third, whereas the division of the study time in before and after DTEP for children attending the center was precise, the definition of corresponding “early” and “late” parts of the study time for children not attending the center, using the mean value of children following DTEP, is somewhat arbitrary. Nonetheless, it is well-recognized that also the observational real-world studies can provide valuable information, especially when their results agree with those of RCTs.

In conclusion, this study supports the hypothesis that a DTEP can contribute to a better control of asthma symptoms in children, especially those with a poor control of their disease, reducing the rate of emergency room access and determining a more efficient use of asthma medicines.

## Data Availability Statement

The raw data supporting the conclusions of this article will be made available by the authors, without undue reservation, to any qualified researcher.

## Ethics Statement

The study was reviewed and approved by the Ethics Committee of the Spedali Civili di Brescia, Brescia (Italy), on 15th June 2015 (registration number 2046). Written informed consent to participate in this study was provided by the participants' legal guardian/next of kin.

## Author Contributions

SG, CQ, VG, RS, RL, FD, VB, MM, CS, and RB: study design. SG, CQ, VG, MFe, MFr, and CG: data collection. AF, EZ, VB, and FD: data analysis. SG, CQ, EZ, and FD: writing of the manuscript. All the authors contributed to reviewing and the final approval of the manuscript.

### Conflict of Interest

The authors declare that the research was conducted in the absence of any commercial or financial relationships that could be construed as a potential conflict of interest.
