# Supplementary material for: Evaluation of a Diagnostic Therapeutic Educational Pathway for Asthma Management in Children and Adolescents
Source: Front Pediatr. 2020 Mar 11;8:39. doi: 10.3389/fped.2020.00039 (PMC7078232; doi:10.3389/fped.2020.00039)
Supplement: Supplementary file 1 [file Table_1.docx]

Supplementary Table I: Number and incident rates (IRs) of prescriptions for the most commonly used drugs for asthma treatment in both intervention and control groups, before and after DTEP and early or late time, respectively.

| Drug combinations | | Intervention (DTEP) group | | | | Control (LHA) group | | | |
| --- | --- | --- | --- | --- | --- | --- | --- | --- | --- |
|  |  | Before | | After | | Early | | Late | |
|  |  | No. | IR per 1000 | No. | IR per 1000 | No. | IR per 1000 | No. | IR per 1000 |
| LABA/ LABA plus glucocorticoid/ LABA plus anticholinergics | Salbutamol | 1412 | 1407.2 | 2863 | 995.2 | 7640 | 1617.7 | 29709 | 788.7 |
|  | Fluticasone/Salmeterol | 400 | 398.6 | 481 | 167.2 | 4172 | 491.1 | 13090 | 386.8 |
|  | Budesonide/Formoterol | 28 | 27.9 | 32 | 11.1 | 480 | 56.5 | 1963 | 58 |
|  | Beclomethasone/Formoterol | 13 | 13.0 | 80 | 27.8 | 352 | 41.4 | 3643 | 107.7 |
|  | Momethasone/Formoterol | 0 | 0 | 0 | 0 | 0 | 0 | 0 | 0 |
|  | Fluticasone/Vilanterol | 0 | 0 | 0 | 0 | 0 | 0 | 0 | 0 |
|  | Fluticasone/Formoterol | 0 | 0 | 8 | 8 | 6 | 0.7 | 178 | 5.3 |
|  | Ipratropium bromide/Fenoterol | 0 | 0 | 0 |  | 8 | 0.9 | 16 | 0.5 |
|  | Ipratropium bromide/Salbutamol | 126 | 125.6 | 88 | 30.6 | 826 | 97.2 | 1229 | 36.3 |
|  | Umiclidinium/Vilanterol | 0 | 0 | 0 | 0 | 0 | 0 | 0 | 0 |
|  | Gycopyrronium bromide/Indacaterol | 0 | 0 | 0 | 0 | 0 | 0 | 0 | 0 |
|  | Aclidinium bromide/Formoterol | 0 | 0 | 0 | 0 | 0 | 0 | 0 | 0 |
|  | Others | 136 | 135.5 | 88 | 30.6 | 746 | 158 | 2226 | 59.1 |
| Systemic steroids | Betamethasone | 332 | 330.9 | 384 | 134.0 | 1715 | 363.1 | 4603 | 122.2 |
|  | Dexamethasone | 1 | 1.0 | 0 | 0 | 79 | 16.7 | 408 | 10.8 |
|  | Methylprednisolone | 1 | 1.0 | 3 | 1.0 | 19 | 4.0 | 160 | 4.2 |
|  | Prednisone | 84 | 83.7 | 227 | 78.9 | 626 | 132.6 | 3296 | 87.5 |
|  | Triamcinolone | 0 | 0 | 1 | 0.3 | 4 | 0.8 | 74 | 2.0 |
|  | Hydrocortisone | 0 | 0 | 2 | 0.7 | 2 | 0.4 | 3 | 0.1 |
|  | Cortisone | 0 | 0 | 0 | 0 | 10 | 2.1 | 4 | 0.1 |
|  | Deflazacort | 0 | 0 | 0 | 0 | 0 | 0 | 0 | 0 |
| Inhaled corticosteroids | Beclomethasone | 705 | 702.6 | 717 | 249.2 | 4922 | 1042.2 | 11779 | 312.7 |
|  | Budesonide | 305 | 304.0 | 208 | 72.3 | 1745 | 369.5 | 4949 | 131.4 |
|  | Flunisolide | 147 | 146.5 | 88 | 30.6 | 1033 | 218.7 | 1803 | 47.9 |
|  | Fluticasone | 436 | 434.5 | 2069 | 719.2 | 2612 | 553.1 | 7744 | 205.6 |
|  | Memethasone | 2 | 2.0 | 3 | 1.0 | 2 | 0.4 | 367 | 9.7 |
|  | Ciclesonide | 0 | 0 | 20 | 6.9 | 7 | 1.5 | 536 | 14.2 |
